# Improved emollient use reduces atopic eczema symptoms and is cost neutral in infants: before-and-after evaluation of a multifaceted educational support programme

**DOI:** 10.1186/1471-5945-13-7

**Published:** 2013-05-16

**Authors:** James M Mason, Julie Carr, Carolyn Buckley, Steve Hewitt, Phillip Berry, Josh Taylor, Michael J Cork

**Affiliations:** 1 School of Medicine, Pharmacy & Health, Durham University, Durham, UK; 2 Sheffield Children’s NHS Foundation Trust, Sheffield, UK; 3 Reckitt Benckiser Healthcare UK, Slough, UK; 4 Partizan International, London, UK; 5 Academic Unit of Dermatology Research, School of Medicine & Biomedical Sciences, University of Sheffield, Sheffield, UK; 6 Department of Dermatology, Sheffield Teaching Hospitals NHS Foundation Trust, Sheffield, UK; 7 Department of Infection and Immunity, The University of Sheffield Medical School, Beech Hill Road, Sheffield, S10 2RX, UK

**Keywords:** Atopic eczema, Children, Emollient therapy, Compliance, Symptoms, Measurement, Community study, Health economics, Patient support, Educational support

## Abstract

**Background:**

Parents and carers of children with eczema often underuse emollient therapy, essential to repairing and protecting the defective skin barrier in atopic eczema. Educational interventions delivered by specialist dermatology nurses in hospital settings have been shown to improve emollient use and reduce symptoms of atopic eczema, but benefits of community-based interventions are uncertain. Support and information about appropriate care may often be inadequate for patients and carers in the community.

**Methods:**

A multifaceted educational support programme was evaluated as a method of increasing emollient use and reducing atopic eczema in children. Support provided for parents and carers included an educational DVD, online daily diary and telephone helpline. The before and after study included 136 British children and their parents, providing baseline and 12 week follow-up data while receiving the programme. Measures included emollient use, POEM and PEST scores, and cost of care.

**Results:**

Average emollient use increased by 87.6 g (95% CI: 81.9 to 119.5 g, p = 0.001) from baseline with the change being immediate and persistent. The POEM score reduced on average by 5.38 (95% CI: 4.36 to 6.41, p = 0.001), a 47% reduction from baseline. Similarly the PEST score reduced on average by 0.61 (95% CI: 0.47 to 0.75, p = 0.001), a 48% reduction from baseline. Sleep disturbance was reduced by 1.27 nights per week (95% CI: 0.85 to 1.68, p = 0.001) and parental feeling of control improved by 1.32 points (95% CI: 1.16 to 1.48, p = 0.001). From the NHS perspective, the programme was cost neutral overall within the study period.

**Conclusion:**

A community-based multifaceted educational support programme greatly increased emollient use, reducing symptoms of atopic eczema and general practitioner contacts, without increasing cost. Significant benefits may accrue to the families and carers of children with atopic eczema due to improved sleep patterns and greater feeling of control. PEST, a new simple measure of acute and remitting atopic eczema severity designed to help parents and children to monitor and manage eczema, merits further evaluation.

## Background

Atopic eczema (synonym atopic dermatitis) in children is unpredictable in its course and may have a profound impact on the quality of life [1-3] of patients and their family, as well as being time-consuming for healthcare professionals to manage. However, the primary care setting is not often well equipped for patient education and support. Twenty years ago, in 1993 a survey of members of the National Eczema Society asked what members wanted healthcare professionals to do to improve the control of childhood atopic eczema [4]. The majority wanted more time to be spent explaining the nature of eczema and advice about how to use the treatments prescribed. In 83% of consultations with general practitioners (GPs) and 74% of first consultations with a dermatologist, the expectations of parents/patients had been only partially met or not met at all.

Education of parents and children with atopic eczema is now recognised as one of the most important interventions in the management of atopic eczema [1,5-10]. The largest RCT of an education programme was conducted in Germany, including 823 children or adolescents with atopic eczema and their families [7]. A six-week education programme for the management of moderate to severe atopic eczema was evaluated, with dermatological, nutritional and psychological facets; it was delivered as a two hour, once-weekly session by a multi-professional team. At one year, improvement in the severity of atopic eczema (SCORAD) in children who received the education programme was significantly greater than in the control group. There were also significant improvements in subjective assessments of severity, itching behaviour and emotional coping in the group receiving education compared to control.

A cost-effectiveness analysis was performed using the data from the German trial as part of the NICE guidelines for treatment of atopic eczema [2]. This demonstrated that if an atopic eczema education programme, similar to that detailed in the German RCT could be provided at less than about £800 per child, then it would be highly likely to be cost effective. NICE also concluded that if a less-resource intensive (and less effective) programme could be implemented in the NHS then this was also likely to be cost-effective.

The impact of specialist dermatology nurse-led education has been evaluated in children with mild/moderate atopic eczema [5,6,11]. These studies showed a significant improvement in the control of atopic eczema in children receiving education. For example Cork and colleagues [6] found that 24% of children with atopic eczema were receiving no emollient treatment, with an average use of emollient of just 54 g per week. Dermatology clinic-based specialist nurse-led educational intervention resulted in an 800% increase in the use of emollients with a corresponding 89% reduction in the severity of the atopic eczema. Although much less resource intensive than the German RCT, these studies were in populations of children with less severe, mild/moderate atopic eczema.

There have been no comparisons of different education programmes for atopic eczema in children apart from a recent comparison of face-to-face care and an e-health intervention [12]. Children (with their parents) and adults with moderate severity atopic eczema attended for a first consultation with a dermatologist and specialist dermatology nurse. Subsequently, they were randomised to face-to-face follow-up treatment in the dermatology department or to internet-guided monitoring and on-line self-management training. There were no significant differences in severity of the atopic eczema, quality of life and intensity of itching between the two groups. Compared to routine care, the e-health intervention led to non-significant changes in health and broader societal costs although estimates were imprecise.

Atopic eczema arises as a result of gene-environment interactions leading to a defective skin barrier [13-15]. Thus, emollient creams, ointments and wash products are the first line treatment to repair the skin. In mild atopic eczema, effective management consists of complete emollient therapy plus occasional treatment of flares with mild potency topical corticosteroids [2,16]. Educational interventions for atopic eczema are provided most commonly in the secondary care hospital setting, while the large majority of children have mild atopic eczema and are treated in primary care [17,18]. Guidelines emphasize the importance of using sufficient emollient: 250 to 500 grams per week [2], the more emollient being used the less the need for mild potency topical corticosteroids [5]. However, the actual use of emollients in the UK is far less than this recommended amount.

Rather than designing an intervention that influenced the selection of prescribed products, we designed a study to assess and improve emollient use and outcomes in children who had currently been prescribed a proprietary emollient, E45 Cream (Reckitt Benckiser Healthcare, UK). Using this design, the amount of emollient could be tracked, along with co-treatment over a 3-month period, allowing the support programme to be assessed. The support programme used a multifaceted approach since evidence from a number of fields supports an integrated, supportive approach for patients as more effective than single or simple measures in achieving behavioural change [19-21].

## Methods

### Objective

To investigate the effectiveness of a multifaceted educational support programme to increase emollient use and reduce atopic eczema symptoms in children.

### Design

Using a before and after study design, a purpose-designed multifaceted educational support programme (ESP) was provided for parents or carers of children with atopic eczema. The programme included an educational DVD, easy-to-use diaries to record eczema condition and daily use of emollients, and telephone support line with dermatology nurses provided regular and on-demand phone support. Data were collected in a 2-week baseline period and 12-week follow-up period (3 months in all). Parents and carers were also asked to recall use of health services in the 12 weeks prior to starting the programme.

### Study population

Parents of children were identified by the Bounty**^®^** Database system operating in the United Kingdom, and sent an email introducing the study. Those interested in participating contacted a free telephone call centre number for further details and to assess eligibility. Parents providing informed consent verbally were provided with a link to the study website and the child’s GP was notified of their participation in writing. The study website provided a second confirmatory electronic consent and access to a baseline diary.

Eligible children were male or female aged 3 months to 6 years; with mild to moderate atopic eczema; and, currently using E45 Cream as their primary emollient.

Children were ineligible where there was a planned absence from home for more than 21 days during the study period; where parents were unable to complete the patient diaries or questionnaires; if receiving systemic medication (e.g. Ciclosporin A, methotrexate) or UV light treatment for their atopic eczema in the 3 months preceding the study; if receiving oral steroids or any new atopic eczema-specific treatment regimen in the 4 weeks preceding the study.

In the original design, referral in the preceding 3 months to a Dermatologist, a Specialist Dermatology Nurse or a GP with a Specialist Interest in Dermatology or receiving specific atopic eczema education or training was an exclusion criterion. However it was not possible pragmatically to exclude patients with a prior visit to an eczema specialist and these were subsequently included. The analysis plan was modified prospectively to include these subjects, with the effect of inclusion explored by sensitivity analysis.

Discussion with the National Research Ethics Service (NRES) established that Independent Ethics Committee approval was not required. The study was considered to be non-interventional service evaluation assessing patient support as an aid to ensure use of emollient according to NICE recommendations and thus not requiring regulatory approval. Products prescribed to patients were not influenced by the study, only the frequency of their use.

### Multifaceted educational support programme

Parents or carers (‘parents’) reported baseline data (weeks -2 to 0) on the study website. On completion of the baseline assessment, parents received by mail an introductory support pack designed to educate, motivate and correct the use of emollient therapy, followed by the first of several telephone interviews and counselling sessions by a dermatologist nurse specialist. Depending on the support needs of the family, further telephone support calls were made on demand, along with optional SMS messaging reminding parent or carers of the patient support eczema management regime.

The support pack included an instructional DVD on the use of emollients in eczema management, featuring the lead dermatology nurse; a booklet on eczema management co-written with the National Eczema Society, London; a hooded towel designed for child use after bathing to improve the experience of emollient application, and a set of daily diaries. Each diary covered a 4-week period and the total follow-up period covered was 12 weeks. On completion, diaries were returned to the agency conducting the study (Partizan International, London, UK). Components of the support programme are available online [22].

During the study, prescribing of E45 Cream by the child’s GP continued in accordance with routine clinical practice. Parents were encouraged to use E45 Cream by depressing the pump 3 times (approximately 12 g), three times a day, or the equivalent for non-pump packs which is 3 level teaspoons (approximately 12 g), three times a day. If followed, this advice would provide approximately 250 g per week. Parents were advised repeatedly to avoid soap and all harsh detergent-based products, replacing these with emollient wash products.

### Outcomes

Baseline and follow-up measures included the following:

***Emollient use (grams per week)*** was estimated in an initial telephone questionnaire based on the emollient pack weight and how long this would usually last. This provided the only estimate of baseline use, in order to avoid the risk of the parent altering their pre-programme practice. Once the support programme had begun, emollient use was captured (as at baseline) at each 4-weekly telephone assessment. Additionally, the number of pumps of emollient used daily was recorded in diaries and the weekly use estimated directly.

***Severity of eczema*** was captured using the POEM (Patient Oriented Eczema Measure) [23]. This recorded the days in a week affected by seven signs of eczema: dryness, itching, bleeding, weeping, flaking, cracking of skin, and by sleep loss.

**Figure 1:**
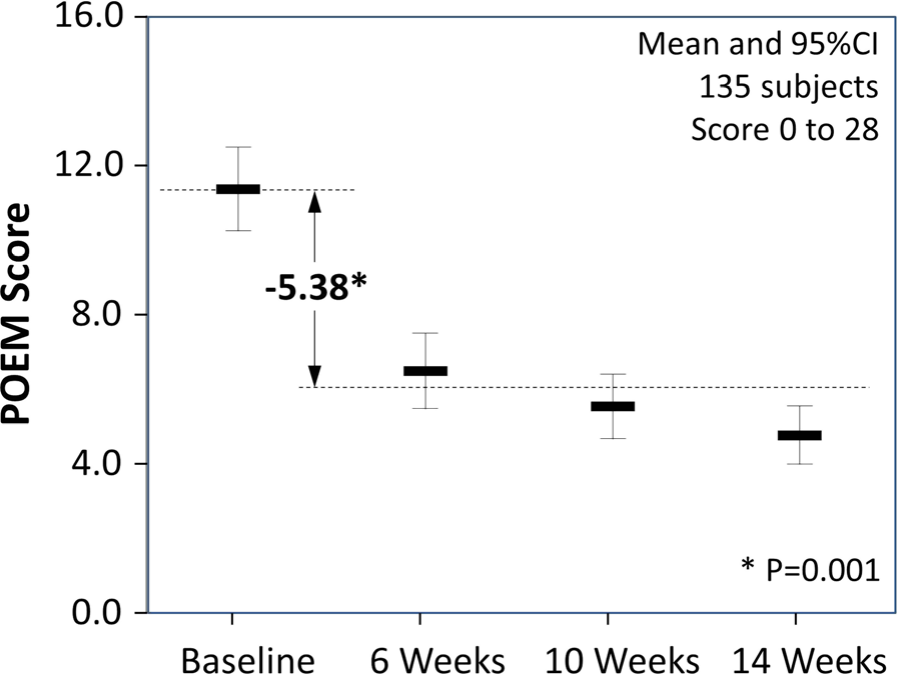
Emollient use before and during the intervention.

Additionally a new simple measure called the Patient Eczema Severity Time (PEST) score was developed for this study, reflecting observations in the clinic that patients’ own summative severity perceptions very closely correlated with conventional severity scores. Thus a simple daily score of ‘overall unhappiness’ with eczema might provide a form of monitoring and feedback to patients and their carers as well as sensitively integrating the sum of eczema experience over time, in a condition with relapsing and remitting severity patterns. The daily diary provided pictorials for users ranging from ‘not at all unhappy’ to ‘extremely unhappy’ scoring 1 to 5 respectively. The PEST score was also designed to be easy for parents to assess in patients too young to vocalise this for themselves (see Figure 1).

***Healthcare contacts.*** The number of GP visits by patients in relation to their eczema was recorded; in the 12 weeks prior to and during the 12 weeks of the programme. Eczema-related visits to a dermatology specialist were recorded during the programme period.

***Parent measures.*** The level of control the parent felt in managing the child’s eczema was captured by telephone questionnaire at baseline and during the programme phase. It was scored from 1 to 5, with 1 being ‘not in control’ to 5 ‘fully in control’.

***Concurrent medication***. Telephone surveys recorded any concurrent treatments used during baseline and the 12 week follow-up, with particular attention to topical corticosteroid use.

### Statistical analysis

There were no reliable estimates for the underuse of emollient and thus potential for improvement in the study population, consequently no formal power calculation was performed. The per-protocol intention was to recruit 150 eligible children age 3 months to 6 years. Given the matched (before-after) data design this would give adequate study power to find small standardized effects in continuous measures (post-hoc: 90% power to detect at effect size of 0.27).

Summary measures were reported for study measures at baseline and in each of the follow-up weeks. Average values were estimated for the 12 week follow-up period to reflect the average effects of the patient support programme and to assess the before-after effect. Changes in paired continuous measures were estimated using the bootstrapping method (with 1,000 replications) to avoid parametric assumptions and changes in paired proportions were evaluated using Fisher’s exact test. Patients with complete data at all points contributed to average period values and differences.

### Economic analysis

Incremental within-study cost analysis was performed from the NHS perspective using nationally reported unit costs for resource items for 2011. The emollient (E45 Cream POM 500 g) was costed at the average English Prescription Pricing Authority-reimbursed rate of £4.89 [24]; GP visits were costed at £36 per visit [25] [D]. The cost of providing the ESP programme was estimated to be £32 per child based on a resource analysis of providing the service.

## Results

### Study population

Programme diaries were completed by 136 British children between August 2011 and March 2012. Subjects were evenly split as Caucasian and non-Caucasian and all parents spoke adequate English. One child was excluded as they did not provide any baseline data, leaving 135 children evaluable. Of these, 18 subjects visited an eczema specialist in the three months prior to joining the programme and thus might have already received additional education on eczema management: these were excluded in a sensitivity analysis.

During the baseline period (-2 to 0 week) the average weekly use of emollient was 79 g (range 4 g to 700 g) (see Table 1). Just 4 children (3%) received at least the NICE minimum dose of 250 g and 12.9% received 125 g/wk. The baseline POEM score was 11.3 (SD: 6.3), and PEST score 2.3 (SD 0.8), 40% and 32% of maximum on each respective scale. Children experienced dryness, redness and itching on average 5.7, 4.1 and 4.7 days a week. Sleep disturbance occurred on average 2.4 nights a week with 21% of parents reporting sleep disturbance at least one night in two. Parental sense of feeling in control of eczema scored an average of 3.3 where 22% of parents reported low levels of control (scoring 1 or 2).

**Table 1:** Changes in emollient use and measures of eczema severity

	**Baseline**^**1**^			**1-4 weeks**			**5-8 weeks**			**9-12 weeks**			**Mean change**^**2**^			
	**Mean**	**(SD)**	**N**	**Mean**	**(SD)**	**N**	**Mean**	**(SD)**	**N**	**Mean**	**(SD)**	**N**	**Mean**	**95% CI**^**3**^	***N***	**p**^**3**^
**Emollient Use (g/wk)**^**4**^																
Daily diary	-			165.2	(96.4)	135	168.4	(113.2)	135	167.8	(109.5)	135	87.6	(81.9 to 119.5)	132	0.001
Time to use	79.2	(79.2)	132	173.5	(114.3)	122	195.2	(112.1)	128	197.4	(106.5)	129	110.0	(94.6 to 131.3)	115	0.001
**Severity Scores**																
POEM^5^	11.34	(6.27)	135	7.52	(5.71)	135	5.50	(5.05)	135	4.85	(5.04)	135	-5.38	(-6.41 to -4.36)	135	0.001
PEST^6^	2.26	(0.81)	135	1.82	(0.73)	135	1.59	(0.78)	135	1.53	(0.68)	135	-0.61	(-0.75 to -0.47)	135	0.001
**Individual Scores**																
Dryness [A] ^7^	5.66	(1.97)	135	4.09	(2.38)	135	3.22	(2.45)	135	2.86	(2.39)	135	-2.28	(-2.67 to -1.90)	135	0.001
Redness [B] ^7^	4.12	(2.44)	135	2.74	(2.42)	135	1.95	(2.18)	135	1.82	(2.13)	135	-1.95	(-2.37 to -1.53)	135	0.001
Itchiness [C] ^7^	4.73	(2.54)	135	3.38	(2.56)	135	2.63	(2.67)	135	2.37	(2.53)	135	-1.94	(-2.36 to -1.52)	135	0.001
[A] + [B] + [C]	14.51	(5.64)	135	10.21	(6.35)	135	7.79	(6.24)	135	7.04	(6.24)	135	-6.17	(-7.17 to -5.20)	135	0.001
Sleep disturbance ^7^	2.36	(2.44)	135	1.49	(2.12)	135	0.98	(1.62)	135	0.80	(1.59)	135	-1.27	(-1.68 to -0.85)	135	0.001
Bleeding^7^	1.34	(1.99)	135	0.68	(1.35)	135	0.42	(1.05)	135	0.33	(1.03)	135	-0.86	(-1.18 to -0.56)	135	0.001
Weeping or oozing^7^	0.86	(1.74)	135	0.40	(1.15)	135	0.24	(0.87)	135	0.18	(0.76)	135	-0.59	(-0.87 to -0.35)	135	0.001
Cracking^7^	2.44	(2.50)	135	1.41	(1.90)	135	1.02	(1.79)	135	0.89	(1.81)	135	-1.34	(-1.77 to -0.97)	135	0.001
Flaking^7^	2.26	(2.63)	135	1.37	(1.96)	135	0.80	(1.47)	135	0.76	(1.57)	135	-1.28	(-1.69 to -0.85)	135	0.001
Roughness^7^	5.49	(2.07)	135	4.25	(2.40)	135	3.32	(2.54)	135	3.56	(2.10)	135	-1.93	(-2.33 to -1.55)	135	0.001
Severity^7^	4.65	(5.16)	135	2.49	(3.86)	135	1.68	(3.17)	135	1.39	(3.02)	135	-2.80	(-3.63 to -2.03)	135	0.001
**Other**																
Parental Control^8^	3.30	(0.99)	135	4.43	(0.66)	134	4.61	(0.57)	135	4.81	(0.41)	135	1.32	(1.16 to 1.48)	134	0.001
GP Visits^**1**^	1.90	(2.13)	135	0.46	(0.54)	135	0.25	(0.44)	135	0.13	(0.36)	135	-1.06	(-1.49 to -0.70)	135	0.002
Steroid Prescribed (%)^9^	51/135	(37.8%)		73/135	(54.1%)		82/135	(60.7%)		82/135	(60.7)%		20.8%	(8.9% to 32.1%)	135	0.001

^1 ^Weeks -2 to 0, except GP visits which included the previous 12 weeks to week 0.

^2 ^The mean change is the average of the programme period scores minus the baseline period score in subjects with complete data.

^3 ^Estimated by bootstrapping with 1,000 replications.

^4 ^Emollient use was estimated using two methods: the time to use a reported weight of emollient (500 g); and, a daily diary record of counts of pumps of emollient used by weight; – the latter method was not available for the baseline period.

^5 ^POEM score from 0 to 28, including seven signs of eczema at four levels of frequency in the past week at the end of each period.

^6 ^PEST score, the child’s unhappiness with eczema: score 1 (not at all) to 5 (very unhappy), daily diary score averaged over each period.

^7 ^Number of days in the previous week has the child had this sign of eczema: score 0 to 7 days, at the end of each period.

^8 ^Parental confidence in managing the child’s eczema: score 1 (not being in control) to 5 (being totally in control), at the end of each period.

^9 ^Proportion of children prescribed topical corticosteroids during each period.

### Clinical outcomes

#### Change in emollient use

**Figure 2:**
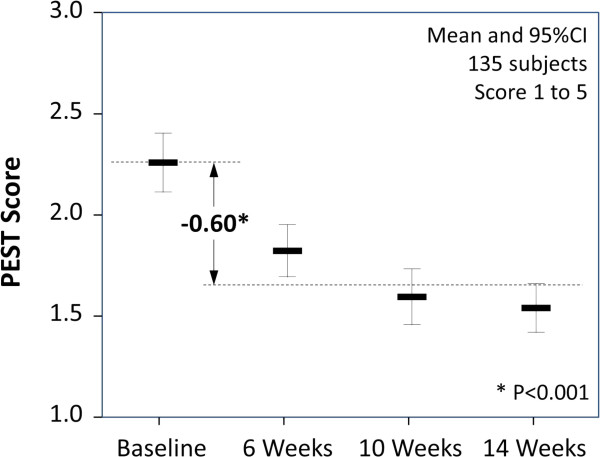
Patient-Orientated Eczema Measure (POEM) before and during the intervention (means and 95% confidence intervals shown).

Diary recorded emollient cream use increased significantly during the 12 weeks of the programme (see Figure 2 and Table 1). On average emollient use increased by 87.6 g (95% CI: 81.9 to 119.5, p = 0.001) with the change being immediate and persistent with 8.9% of children receiving 250 g/wk at 12 weeks and 61.5% receiving 125 g/wk.

Emollient use was also estimated, as at baseline, as the time taken in each period to use a prescribed emollient cream pack of known weight. The estimated increase in emollient was higher at 110 g/wk (95% CI: 94.6 to 131.3, p = 0.001) although only 85% of parents reported this measure.

From a baseline of 37.8% of patients, prescription of topical corticosteroids increased significantly by 20.8% (95% CI: 8.9% to 32.1%, p = 0.001) during the programme. The volume of use of steroid was not recorded but hydrocortisone 1% accounted for 70% of total use and clobetasone butyrate 0.05% (in various preparations) for 25%. Although topical steroids were prescribed, parents reported that they used these in minimal quantities due to concerns about side effects. An increase in topical steroid use was not planned as part of the educational support provided, but often arose in telephone support sessions.

#### Changes in measures of eczema severity

**Figure 3:**
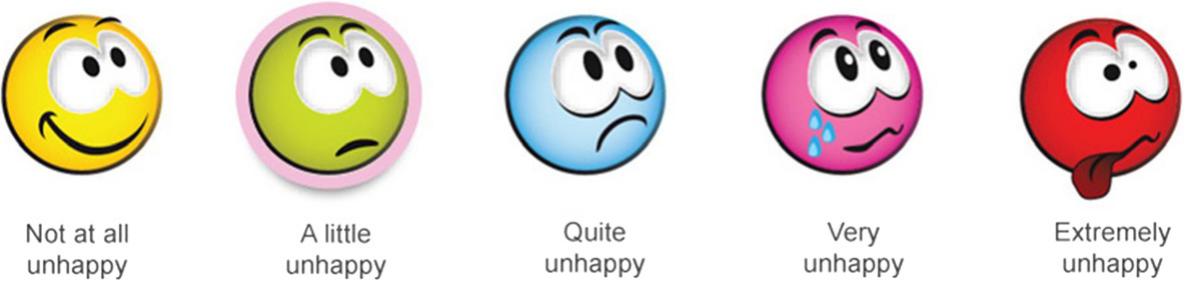
Patient Ezcema Severity-Time Score (PEST) before and during the intervention (means and 95% confidence intervals shown).

Eczema severity reduced significantly during the 12 weeks of the programme. The POEM score reduced on average by 5.38 (95% CI: 4.36 to 6.41, p = 0.001), a 47% reduction from the baseline score (see Figure 3 and Table 1). Individual signs of eczema consistently followed the pattern of improvement seen in the aggregated POEM score (see Table 1).

**Figure 4:**
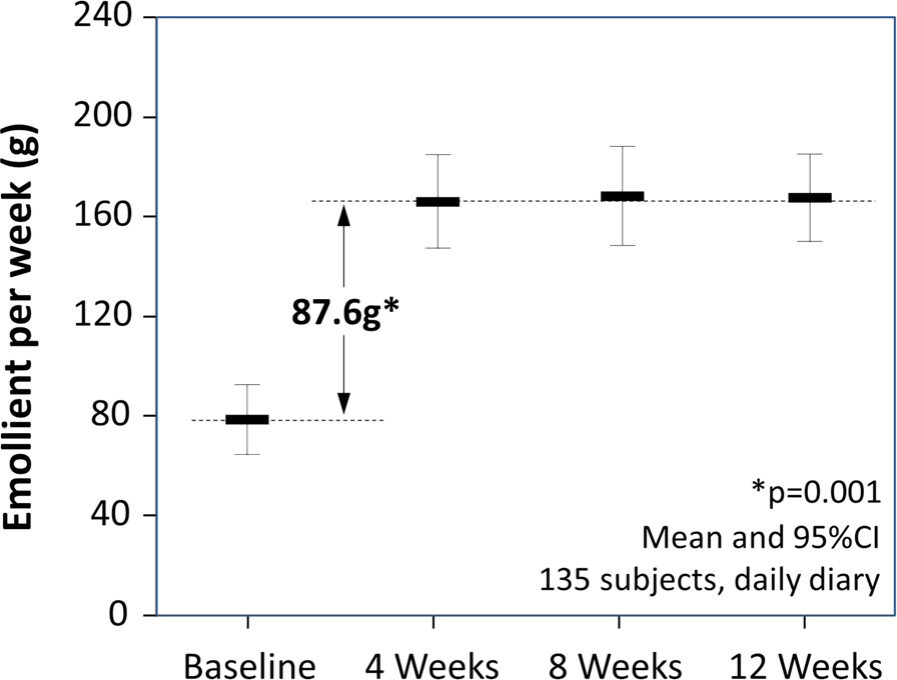
Patient Ezcema severity-time score (PEST) graphic.

Similarly the PEST score reduced on average by 0.61 (95% CI: 0.47 to 0.75, p = 0.001), a 48% reduction from the baseline score (see Figure 4 and Table 1). During the programme, 45.9% of children were reported as having an average POEM score of 0 to 2 compared to 4.4% at baseline; similarly 56.3% of children were reported with a PEST score of 1 compared to 13.3% at baseline. POEM and PEST scores were strongly correlated with Pearson correlation coefficients at baseline, 4, 8, and 12 weeks of 0.56, 0.51, 0.63 and 0.71 (P < 0.01 in all instances).

Of particular note are two measures which may have broader implications for the families of children suffering with eczema. Loss of sleep if persistent may have significant knock-on consequences for the health and well-being of family members. On average, the number of nights per week experiencing sleep disturbance was reduced during the 12 week programme by 1.27 nights (95% CI: 0.85 to 1.68, p = 0.001), a halving of the baseline level of disturbance. Secondly, parental feeling of control of their child’s eczema improved by 1.32 points (95% CI: 1.16 to 1.48, p = 0.001) with 91.5% of parents reporting the highest level of control.

### Health economic outcomes

Costs of care in the periods preceding and during the patient support programme are tabulated in Table 2. There was a statistically significant increase in the cost of emollient by about £10 using the diary method, or about £13 using the ‘time-in-use’ method. GP visits fell on average by about 1 visit per child (Table 1) leading to no overall or significant change in net cost. The finding was similar regardless of the method of estimating emollient use.

**Table 2:** Costs of care in the baseline and programme periods

	**Pre-Programme**^**1**^		**Programme**^**2**^		**Change**^**3,4**^			
	**Mean**	**(SD)**	**Mean**	**(SD)**	**Mean**	**95% CI**^**4**^	**N**	**p**
**Cost (£, 2011)**								
Programme			32.00	-	32.00	-	-	-
Emollient [A]	9.29	(9.30)	19.57	(12.03)	10.28	(7.93 to 12.64)	132	0.001
Emollient [B]	9.72	(9.69)	22.64	(12.21)	12.91	(10.72 to 15.10)	115	0.001
GP visits	68.53	(76.70)	30.40	(31.67)	-38.13	(-52.58 to -23.68)	135	0.001
Overall cost [A]	78.29	(79.34)	82.66	(34.16)	4.37	(-10.55 to 19.30)	132	0.62
Overall cost [B]	80.32	(82.48)	84.37	(31.29)	4.06	(-12.00 to 20.11)	115	0.56

^1 ^Estimated for the 12 weeks preceding intervention, using 2 week baseline data and 12 week recall (GP visits).

^2 ^Twelve weeks while receiving the patient support programme.

^3 ^Difference in costs in the two periods (negative denotes a reduction).

^4 ^Estimated by bootstrapping with 1,000 replications.

A Emollient use costed using the daily diary method.

B Emollient use costed using the estimated time taken to use a 500 g pot of emollient.

### Sensitivity analysis

Analyses were repeated in 117 subjects, excluding 18 subjects who had visited an eczema specialist in the three months prior to joining the programme. Qualitatively, findings were unchanged in this subgroup (Table 3).

**Table 3:** Sensitivity analysis: excluding children who had recently seen a specialist

	**Pre-Programme**^**1**^		**Programme**^**2**^		**Change**^**3,4**^			
	**Mean**	**(SD)**	**Mean**	**(SD)**	**Mean**	**95% CI**^**4**^	**N**	**p**^**4**^
**Emollient Use (g/wk)**								
Daily diary	69.7	(49.5)	170.3	(99.0)	100.6	(81.9 to 119.5)	114	0.001
Time to use	72.2	(49.6)	184.3	(97.6)	112.2	(94.6 to 131.3)	99	0.001
**Severity Scores**								
POEM	11.03	(6.49)	5.83	(4.67)	-5.20	(-6.39 to -4.34)	117	0.001
PEST	2.26	(0.82)	1.66	(0.62)	-0.60	(-0.74 to -0.47)	117	0.001
**Cost (£, 2011)**								
Programme			32.00	-	32.00	-	-	-
Emollient [A]	8.18	(5.80)	19.98	(11.61)	11.81	(9.81 to 14.01)	114	0.001
Emollient [B]	8.47	(5.82)	21.63	(11.46)	13.16	(10.94 to 15.39)	99	0.001
GP visits	63.23	(63.80)	30.46	(30.86)	-32.77	(-45.77 to -19.77)	117	0.001
Overall cost [A]	71.81	(66.37)	83.25	(33.75)	11.44	(-1.99 to 24.86)	114	0.123
Overall cost [B]	72.11	(68.24)	83.45	(33.75)	11.35	(-3.14 to 25.83)	99	0.239

^1 ^Estimated for the 12 weeks preceding intervention, using 2 week baseline data and 12 week recall (GP visits).

^2 ^Twelve weeks while receiving the patient support programme.

^3 ^Difference in costs in the two periods (negative denotes a reduction).

^4 ^Estimated by bootstrapping with 1,000 replications.

A Emollient use costed using the daily diary method.

B Emollient use costed using the estimated time taken to use a 500 g pot of emollient.

## Discussion

Given the considerable underutilisation of emollients for atopic eczema in children, effective training for parents in the use of topical therapy is a key issue. Our experience within the dermatology clinic has underlined the importance of a comprehensive approach to support and education, including printed, verbal and visual components to achieve the broadest effect [6]. The study findings demonstrate the potential utility of providing a patient-centric support package to enhance concordance with treatment goals and improve patient outcomes.

Primary care practitioners are sometimes concerned about the practicality of delivering multifaceted interventions in routine care. The current study demonstrates that a coherent multifaceted programme, delivered at a distance using a specialist dermatology nurse, may be a cost-neutral use of NHS resources. Primary care commissioners might consider an appropriate local adaptation of the ESP to address patients’ needs, while accepting the need not to subtract from the components, which may work synergistically. Details of the ESP are available online to assist those developing services [22]. Education regarding the use of topical products including emollients is an essential part of the management of many other skin diseases such as psoriasis [26]. Similar ESPs to that evaluated in this study for atopic eczema could enhance adherence with topical treatment regimens and improve outcomes in these skin diseases.

Topical steroid prescriptions increased during the study, although it is unlikely that steroid use profoundly affected the study findings. In common with our clinical experience, parents in the study reported using steroids minimally and change in availability only occurred in 20% of patients. However, volume of use of prescriptions was not measured formally.

Two measures within the study captured aspects of the impact of childhood eczema on the broader family. Increased emollient use was significantly related to reduced sleep disturbance and greater sense of parental control through a greater understanding of the disease and their child’s symptoms. Parents expressed frustration at the inconsistency of information and advice provided in primary care about eczema and its management. A qualitative analysis of patient narratives will be published separately.

Within the duration of the programme the overall cost was cost-neutral while providing tangible health benefits to children and their families. It is likely that cost savings would continue to accrue beyond the duration of the study hence the within-programme analyses presented should be viewed as conservative. If savings are extrapolated then, under a range of assumptions, the ESP is likely be both cost saving and symptom-reducing in children with mild to moderate atopic eczema.

### Limitations

While introduction of the ESP provided a strong contemporaneous improvement in emollient treatment and subsequent atopic eczema symptoms, the study design lacked the protection against bias afforded by a randomised controlled design. The major threat to attribution is regression to the mean where a concerned sub-group of parents enrol children with naturally recurring and remitting eczema at a point of acute symptoms, which tend naturally to lessen. A control group selected by randomisation would separate out programme and regression effects. However the changes in emollient use and in measures of eczema severity are dramatic, consistent and contemporaneous with the programme and are thus likely to preclude a pure regression effect. A further threat lies in the generalizability of findings if a highly selected population with atypical characteristics (in the parent or child) have volunteered to participate in the study, although this is mitigated by the broad inclusion by ethnicity and socioeconomic group. Finally, parents were asked to recall visits to primary care and specialists in the 12 weeks preceding baseline, a long period of recall potentially introducing recall bias.

## Conclusions

The educational support programme (ESP) provided coherent messaging and support to parents and their children with atopic eczema. During the 12 week course of the programme there was a dramatic and significant increase in emollient use and also a small associated increase in mild potency steroid use. The ESP dramatically reduced signs of eczema, sleep disturbance and parental feelings of lack of control. Analysis at 12 weeks provides evidence that the ESP is a cost-neutral strategy although this will be further explored at 12 months. A new, simple measure of eczema severity (PEST score) designed to help parents and children to monitor and manage eczema showed similar sensitivity and high correlation with the POEM [23] measure, and thus merits further evaluation.

## Competing interests

The research was funded by an educational grant to participating academic and NHS institutions from Reckitt Benkiser Healthcare. Partizan International is an agency specialising in patient support programmes.

JM Mason received institutional funding from Reckitt Benckiser Healthcare UK to support his participation in the study and attendance at BAD, Birmingham 2012.

MJ Cork has acted as a consultant and has given lectures for Reckitt Benckiser Healthcare UK.

C Buckley, S Hewitt and P Berry are employees of Reckitt Benkiser Healthcare UK, which makes and distributes the emollient product provided to patients in this study.

## Authors’ contributions

JMM provided methodological, statistical and economic oversight to the study. He led the analyses and writing of the paper. MJC provided study concept, design, clinical and methodological oversight. He participated in the analysis and writing and interpretation of the findings. CB, SH and PB are employees of RB Healthcare UK. All contributed to the study design, writing and interpretation of the findings. JT led the service conducting telephone interviews. He contributed to the study design, writing and interpretation of the findings. All authors read and approved the final manuscript.

## Authors’ information

Professor Michael Cork is Head of Academic Dermatology Research in the Department of Infection & Immunity at The University of Sheffield Medical School and Honorary Consultant Dermatologist to both Sheffield Children’s and Teaching Hospitals. He has published extensively in basic and applied science with particular research interests in atopic dermatitis (atopic eczema), psoriasis, alopecia areata and vitiligo. Internationally, his unit is one of the leading groups translating basic dermatological science into new treatments, including ’Skin Protease Inhibitors’ and ’Vitamin A Metabolic Pathway Inhibitors.

Professor James Mason is a health economist for Durham University who has published extensively using health economics, health service research and statistical methodologies. He is health economist for a number of ongoing and published dermatological trials. He is Director of Durham Clinical Trials Unit and co-Director of the Research Design Service for the North East.

## Pre-publication history

The pre-publication history for this paper can be accessed here:

http://www.biomedcentral.com/1471-5945/13/7/prepub
